# Special Issue “Recent Advances in Medicinal Plants and Natural Products”

**DOI:** 10.3390/ijms27031350

**Published:** 2026-01-29

**Authors:** Ângelo Luís, Ana Paula Duarte

**Affiliations:** RISE-Health, Department of Medical Sciences, Faculty of Health Sciences, University of Beira Interior, Av. Infante D. Henrique, 6200-506 Covilhã, Portugal

Medicinal plants and natural products continue to represent a rich and rapidly evolving field in molecular sciences. Their longstanding importance in traditional medicine, coupled with modern analytical and biotechnological advancements, has accelerated scientific interest in uncovering novel therapeutic agents from natural sources. The Special Issue “Recent Advances in Medicinal Plants and Natural Products,” published within the *Bioactives and Nutraceuticals* section of the *International Journal of Molecular Sciences (IJMS)*, was designed to highlight this dynamic research landscape and promote interdisciplinary discussions around natural bioactive compounds. As outlined in the description, this Special Issue showcases studies exploring the therapeutic potential of natural compounds, advances in extraction and chemical characterization techniques, and molecular investigations that clarify mechanisms of action (Figure 1).

In recent years, major breakthroughs in analytical chemistry, molecular biology, and biotechnology have transformed how natural compounds are studied. High-resolution chromatographic and spectroscopic techniques have facilitated the discovery of new metabolites, while molecular approaches have illuminated the mechanisms by which these substances exert antioxidant, antimicrobial, and other bioactivities. A distinguishing characteristic of this Special Issue is its strong emphasis on bridging fundamental molecular insights with applied research. The scope explicitly called for contributions that deepen the understanding of molecular mechanisms behind biological activities, allowing researchers to evaluate therapeutic potential through rigorous mechanistic frameworks.

Medicinal plants and natural products remain highly relevant for researchers, clinicians, and policymakers. The contributions collected in this Special Issue demonstrate the broad applicability of natural-compound research across domains ranging from human health and disease management to functional biomaterials and sustainable biotechnologies. In alignment with IJMS editorial values, the Special Issue promotes open-access dissemination, ensuring researchers worldwide can benefit from the findings and engage in global scientific dialog. By offering a curated collection of high-quality, peer-reviewed studies, the Special Issue aims to inform decision-making, stimulate technological development, and reinforce the relevance of natural-products research in modern healthcare and innovation ecosystems.

Natural products continue to occupy a central and enduring role in the search for innovative therapeutic solutions. Their structural diversity, evolutionary optimization, and wide biological activities make them exceptionally rich sources of pharmacologically active molecules. The articles collected in this Special Issue reflect the vibrancy of the field today—combining traditional knowledge, advanced chemical analysis, biological assays, and computational modeling to explore the therapeutic applications and mechanistic complexities of plant-derived compounds.

A recurring theme throughout this Special Issue is the robust characterization and optimization of bioactive compounds. Studies on *Amomyrtus luma* [contribution 1] and *Vaccinium oxycoccos* [contribution 2] provided compelling examples of how phytochemical richness is shaped by environmental factors, ripening stages, and extraction parameters. High phenolic contents, dynamic flavonoid fluctuations, and antioxidant activities reveal how these fruits can be strategically valorized for health-oriented applications. The cinnamaldehyde-rich essential oil of *Cinnamomum zeylanicum* further expands this chemical panorama, displaying strong antioxidant, antimicrobial, immunomodulatory, and antitumor effects [contribution 3]. Meanwhile, the multifaceted chemical composition of *Foeniculum vulgare* extracts—validated through HPLC, GC-MS, and molecular docking approaches—demonstrated their potential as natural antifungal- and antioxidant agents [contribution 4].

Another major axis in this Special Issue is centered on metabolic regulation, inflammation, and tissue homeostasis. Fenugreek extract (Forceterone^®^) showed strong in vivo and in vitro efficacy in reducing hormone-induced prostate enlargement, modulating inflammatory cytokines, and rebalancing apoptotic pathways [contribution 5]. Research on *Viola yedoensis* extract demonstrated its intriguing potential for skin-barrier repair, upregulating CD44, aquaporin-3, filaggrin, and keratin-10, supported by advanced metabolomic profiling that uncovered numerous previously undescribed compounds [contribution 6]. In a metabolic context, licochalcone D from *Glycyrrhiza uralensis* ameliorated glucose dysregulation and restored mitochondrial function in insulin-resistant hepatocytes, offering promising insights into natural compounds for managing metabolic disorders [contribution 7].

Complementing these biological and analytical studies, structure-based and computational methodologies shed light on molecular mechanisms and multi-target interactions. Phytochemicals from *Detarium senegalense*, such as catechin and epicatechin, exhibited strong binding affinities for PDE5 and other enzymes involved in erectile dysfunction, displaying pharmacokinetic and drug-likeness advantages over synthetic PDE5 inhibitors [contribution 8]. Similarly, T-muurolol showed broad antibacterial potential against *Staphylococcus aureus*, with stable interactions across multiple essential bacterial proteins, coupled with antioxidant, anti-inflammatory, and favorable ADMET properties [contribution 9].

The Special Issue concludes with two comprehensive reviews that expand the scope from specific compounds to broader therapeutic and societal implications. Stilbenes—well-known for their cardioprotective, neuroprotective, anti-inflammatory, and anticancer properties—were discussed in depth, with special attention to bioavailability challenges and emerging formulation strategies [contribution 10]. Finally, *Salvia divinorum* was examined from both a pharmacological and public-health perspective, integrating toxicological evidence, therapeutic potential, patterns of recreational use, and global regulatory complexities [contribution 11].

Together, these contributions illustrate a rich and interconnected landscape. From discovery and characterization to mechanistic elucidation, computational refinement, and translational relevance, they reaffirm natural products as essential pillars in the search for safer, more effective therapeutics and highlight the crucial role of interdisciplinary science in shaping the future of phytochemical research.

The Special Issue “Recent Advances in Medicinal Plants and Natural Products” reflects the richness, diversity, and scientific momentum of this field. By bringing together multidisciplinary insights and highlighting innovative methodologies, the collection offers a valuable resource for advancing natural products research. We hope it will inspire continued innovation, foster new collaborations, and contribute to a deeper understanding of nature’s molecular richness as a foundation for future therapeutic development.

## Figures and Tables

**Figure 1:**
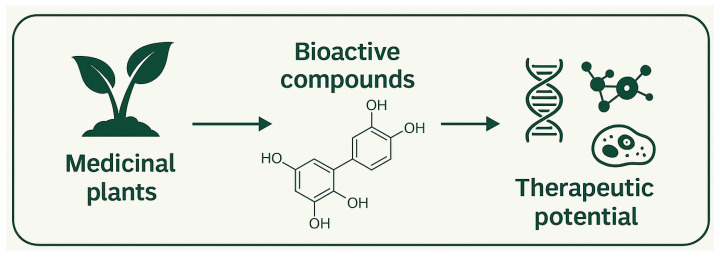
Aims and scope of the Special Issue “Recent Advances in Medicinal Plants and Natural Products.”

## Data Availability

No new data were created.

